# Numerical results for the multiobjective trust region algorithm MHT

**DOI:** 10.1016/j.dib.2019.104103

**Published:** 2019-06-06

**Authors:** Jana Thomann, Gabriele Eichfelder

**Affiliations:** Institute for Mathematics, Technische Universität Ilmenau, Ilmenau, Germany

**Keywords:** Multiobjective optimization, Heterogeneous optimization, Test set, Test problems, Trust region algorithm

## Abstract

In this data article, we report data and numerical results related to the research article entitled ”A trust region algorithm for heterogeneous multiobjective optimization” by Thomann and Eichfelder in SIAM Journal on Optimization. The method MHT which is presented there is designed for multiobjective heterogeneous optimization problems where one of the objective functions is an expensive black-box function, for example given by a time-consuming simulation. Here, we present the data of numerical tests with a set of 78 test problems mainly collected from literature and only complemented by few self-chosen test problems. The presence of expensive functions is artificially introduced in the test problems by defining one of the objective functions as expensive.

**Table:** Specifications table

Subject Area	Mathematics
More specific subject area	Multiobjective optimization
Type of data	Numerical results (numbers, tables, figures) of test runs of optimization algorithm MHT from Ref. [13]
How data was acquired	Output data of the optimization algorithm MHT from Ref. [13]
Data format	Raw, filtered, summarized, analyzed
Experimental factors	Output data of the optimization algorithm MHT was stored, these raw data were filtered and summarized for the presentation in this paper
Experimental features	List of test problems
	Presentation and illustration of results of test runs with algorithm MHT from Ref. [13] and comparison to two other algorithms
Data source location	Ilmenau, Germany, Technische Universität Ilmenau
Data accessibility	Only in this article
Related research articles	J. Thomann and G. Eichfelder. A trust region algorithm for heterogeneous multiobjective optimization. SIAM J. on Optim., 29(2), 1017–1047, 2019 [13]

**Table:** 

**Value of the data** • Output data reported represents a benchmark for future comparisons, among algorithms for heterogeneous multiobjective optimization problems • Output data illustrates how a trust region idea can be used in the context of heterogeneous optimization problems • Output data reported illustrates the behavior of the trust region method MHT and its theoretical properties • Output data illuminates similarities and differences of MHT, the direct search approach DMS and the trust region method EFOS

## 1 Data

We report the data related to the results of the numerical tests of the algorithm MHT presented in Ref. [13]. All computations have been run on an Intel(R) Core(TM) i3-2105 CPU 3.10GHz with Matlab 2017a.

All considered test problems are listed in Table 1 with references, number of objective functions and further information. The output data of MHT is presented structured by convex, nonconvex and scalable test problems. The output data of MHT presented includes starting points, the points that are given as output of MHT and required function evaluations. For some instances also tables with a comparison of used function evaluations are given.

**Table 1:** Test problems.

name	n	constraints	convexity	PF	exp.
bi-objective test problems (q = 2)					
BK1 [1], [7]	2	box	conv.	conv.	$f_{1}$
CL1 [1]	4	box	nonc.	conv.	$f_{1}$
Deb41 [1], [2]	2	box	conv.	conv.	$f_{2}$
Deb53 [1], [2]	2	unc.	nonc.	nonc.	$f_{2}$
Deb513 [1], [2]	2	box	nonc.	discon.	$f_{2}$
Deb521b [1], [2]	2	box	nonc.	nonc.	$f_{2}$
DG01 [1], [7]	1	box	nonc.	conv.	$f_{1}$
DTLZ1 [1], [3]	2	box	nonc.	conv.	$f_{1}$
ex005 [1], [8]	2	box	nonc.	nonc.	$f_{2}$
Far1 [1], [7]	2	box	nonc.	nonc.	$f_{1}$
FF [5]	2,3,4,5	box	nonc.	nonc.	$f_{1}$
Fonseca [6]	2	box	nonc.	nonc.	$f_{1}$
IM1 [1], [7]	2	box	nonc.	conv.	$f_{1}$
Jin1 [1], [9]	2,3,4,5,10,20,30,40,50	box	conv.	conv.	$f_{1}$
Jin2 [1], [9]	2,3,4,5	box	nonc.	conv.	$f_{2}$
Jin3 [1], [9]	2,3,4,5	box	nonc.	nonc.	$f_{2}$
Jin4 [1], [9]	2,3,4,5	box	nonc.	nonc.	$f_{2}$
JOS3 [7]	3	box	conv.	conv.	$f_{1}$
Kursawe [1], [10]	3	box	nonc.	discon.	$f_{1}$
Laumanns [7]	2	box	conv.	conv.	$f_{1}$
LE1 [1], [7]	2	box	nonc.	nonc.	$f_{1}$
Lis [7]	2	box	nonc.	nonc.	$f_{1}$
lovison1 [1]	2	box, unc.	conv.	conv.	$f_{1}$
lovison2 [1]	2	box, unc.	nonc.	nonc.	$f_{2}$
lovison3 [1]	2	box, unc.	nonc.	conv.	$f_{1}$
lovison4 [1]	2	box, unc.	nonc.	nonc.	$f_{1}$
MOP1 [1], [7]	1	unc.	conv.	conv.	$f_{1}$
Schaffer2 [11]	1	box	nonc.	discon.	$f_{1}$
T1	2	unc.	conv.	conv.	$f_{1}$
T2	2	unc.	nonc.	nonc.	$f_{2}$
T3	2	box	conv.	conv.	$f_{2}$
T4	2,3,4,5,10,20,30,40,50	box	conv.	conv.	$f_{1}$
T5	2	box	conv.	conv.	$f_{1}$
T6	2	box	conv.	conv.	$f_{1}$
T7	3	box	conv.	conv.	$f_{1}$
VU1 [1], [7]	2	box	nonc.	conv.	$f_{1}$
VU2 [1], [7]	2	box	conv.	conv.	$f_{2}$
ZDT1 [1], [15]	4	box	nonc.	conv.	$f_{2}$
ZDT2 [1], [15]	4	box	nonc.	nonc.	$f_{2}$
ZDT3 [1], [15]	4	box	nonc.	discon.	$f_{2}$
ZDT4 [1], [15]	2	box	nonc.	conv.	$f_{2}$
ZDT6 [1], [15]	4	box	nonc.	nonc.	$f_{2}$
test problems with q = 3 objective functions					
FES2 [1], [7]	10	box	nonc.	nonc.	$f_{3}$
IKK1 [1], [7]	2	box	conv.	conv.	$f_{1}$
T8	3	box	conv.	conv.	$f_{3}$
ZLT1 [1], [7]	4	box	conv.	conv.	$f_{3}$

The presented figures illustrate the behavior of the algorithm and show, besides the starting point, all iteration points and all further points that are required during the algorithm. Furthermore, there are also figures which show performance profiles of MHT and two other algorithms, DMS [1]and EFOS [12].

In subsection 2.1 an overview of the test problems used for the algorithm in Ref. [13] is presented followed by numerical results in subsection 2.2.

## 2 Experimental design, materials and methods

### 2.1 Test problems

We consider the general optimization problem

(MOP) $$\underset{x\in \text{Ω}}{\text{min}}f\left(x\right)$$

with $\text{Ω}\subseteq R^{n}$ and $f\left(x\right)={\left(f_{1}\left(x\right),\ldots ,f_{q}\left(x\right)\right)}^{⊤}$. The objective functions $f_{i}:R^{n}\to R$ are assumed to be twice continuously differentiable for all i=1,2,…,q and ${\text{max}}_{i=1,\ldots ,q}f_{i}\left(x\right)$ is assumed to be bounded from below. The constraint set Ω contains either box constraints or is the whole domain. In MHT, heterogeneous optimization problems with one expensive function are considered.

The following set of 78 test problems is based on test problems from the literature for general multiobjective and derivative-free algorithms [1], [2], [3], [5], [6], [7], [8], [9], [10], [11], [14], [15] and completed with some self-chosen problems. The latter are listed in subsection 3. All considered problems are test problems and do not involve an actual expensive function. For these problems the efficient points can be computed which is necessary to compare the results of the algorithm to the actual efficient solutions. For evaluating the results one of the functions is declared as expensive and the amount of function evaluations for this function is counted.

Among the test examples are quadratic and nonquadratic functions, convex and nonconvex problems, either unconstrained or with box constraints. Table 1 shows an overview of all 78 considered test problems with information about the dimension of the domain (*n*), the constraints and the convexity of the problem. It also includes information about the geometry of the Pareto front, the set of all nondominated points (convex, nonconvex, disconnected). We say the Pareto front PF is convex (nonconvex) if the set $PF+R_{+}^{2}$ is convex (nonconvex). Besides, the table contains in its last column which of the objective functions is declared as expensive for the test runs of MHT. If there are significant differences regarding the difficulty of the functions, the more difficult function is declared as expensive.

Some of the test examples are scalable and different values for *n* are considered. They are listed in the table. For every test problem several randomly generated, but fixed, starting points were used. One test instance is defined as one test problem with one starting point. In total, 802 test instances have been considered, among them 348 convex and 454 nonconvex instances.

### 2.2 Numerical results

The multiobjective trust region algorithm MHT as presented in Ref. [13] has been implemented in Matlab (version 2017a) and tested for the multiobjective problems with two or three objective functions from subsection 2.1. We used the realization of the trust region update as described in Section 5.2 in [13] with the parameters $\eta_{1}=0.001,\eta_{2}=0.9$. The stopping criterion is implemented according to Section 5.1 in [13]. It uses a maximum number of function evaluations given by the user, the size of the trust region and a necessary condition for local weak efficiency. Furthermore, model information for the as expensive declared function is reused as often as possible to save function evaluations, see Section 5.4 in [13].

We compared MHT with two other methods. On the one hand, since MHT computes only one point fulfilling a necessary optimality criterion and does not approximate the set of efficient points, we used the weighted sum approach with equal weights and apply EFOS (Expensive Function Optimization Solver) [12] to it with the predefined standard parameters. It is a solution method for expensive, simulation-based scalar optimization problems also using the trust region approach. As a stopping criterion a criticality measure using the gradients of the model functions is applied in conjunction with a validity criterion for the models. For convex multiobjective optimization problems every efficient point can be computed by a weighted sum of the objectives with suitable weights. For nonconvex problems only a subset of the efficient points can be computed. This needs to be regarded when comparing the results.

On the other hand, and to circumvent the disadvantages of the weighted sum approach, the multiobjective method DMS [1] is used as a comparative method. It is a direct search approach and therefore derivative-free and suitable for expensive functions. It approximates the whole set of efficient points, but offers also the option to compute only one efficient point. We used the latter option with the predefined standard parameters varying the maximum number of function evaluations. As a stopping criterion DMS uses a maximum number of function evaluations given by the user and the step size for the search step. If the step size is lower than the predefined value $\left(10^{-3}\right)$, DMS stops. Furthermore, DMS includes a method to compute starting points on its own which is chosen by default. To use the starting point the user passes as input to the algorithm, the parameter ‘list’ in the parameter file needs to be changed from the predefined value 3 to 0.

Of course also the way of the implementation of the algorithms influence the performance for the test problems. In the currently available implementation of EFOS often internal errors occur and runs are terminated without having computed a solution.

As a main comparison criterion we use the number of function evaluations until the considered method terminates and set the maximum number of allowed function evaluations for all algorithms to 2000. Firstly, we present some selected test instances to illustrate the procedure of MHT and to compare the results to the methods DMS and EFOS. In the end of this section, we present performance profiles for all considered test instances.

#### 2.2.1 Convex test problems

At first we consider the quadratic, convex test problem (BK1) from Refs. [1], [7] given by

(BK1) $$\underset{x\in \text{Ω}}{\text{min}}\left(\begin{array}{c} f_{1}\left(x\right)\\ f_{2}\left(x\right) \end{array}\right)=\underset{{x\in \left[-5,10\right]}^{2}}{\text{min}}\left(\begin{array}{c} x_{1}^{2}+x_{2}^{2}\\ {\left(x_{1}-5\right)}^{2}+{\left(x_{2}-5\right)}^{2} \end{array}\right)$$

to illustrate the procedure of MHT. For this test problem function $f_{1}$ is declared as expensive function. For all instances of this test problem MHT and EFOS compute efficient points, DMS only for most of the instances. EFOS computes for different starting points always the same efficient point, whereas MHT and DMS generate different efficient points. MHT needs 12–13 expensive function evaluations and therefore significantly less than EFOS (57–73) and DMS (41–61).

**Fig. 1:**
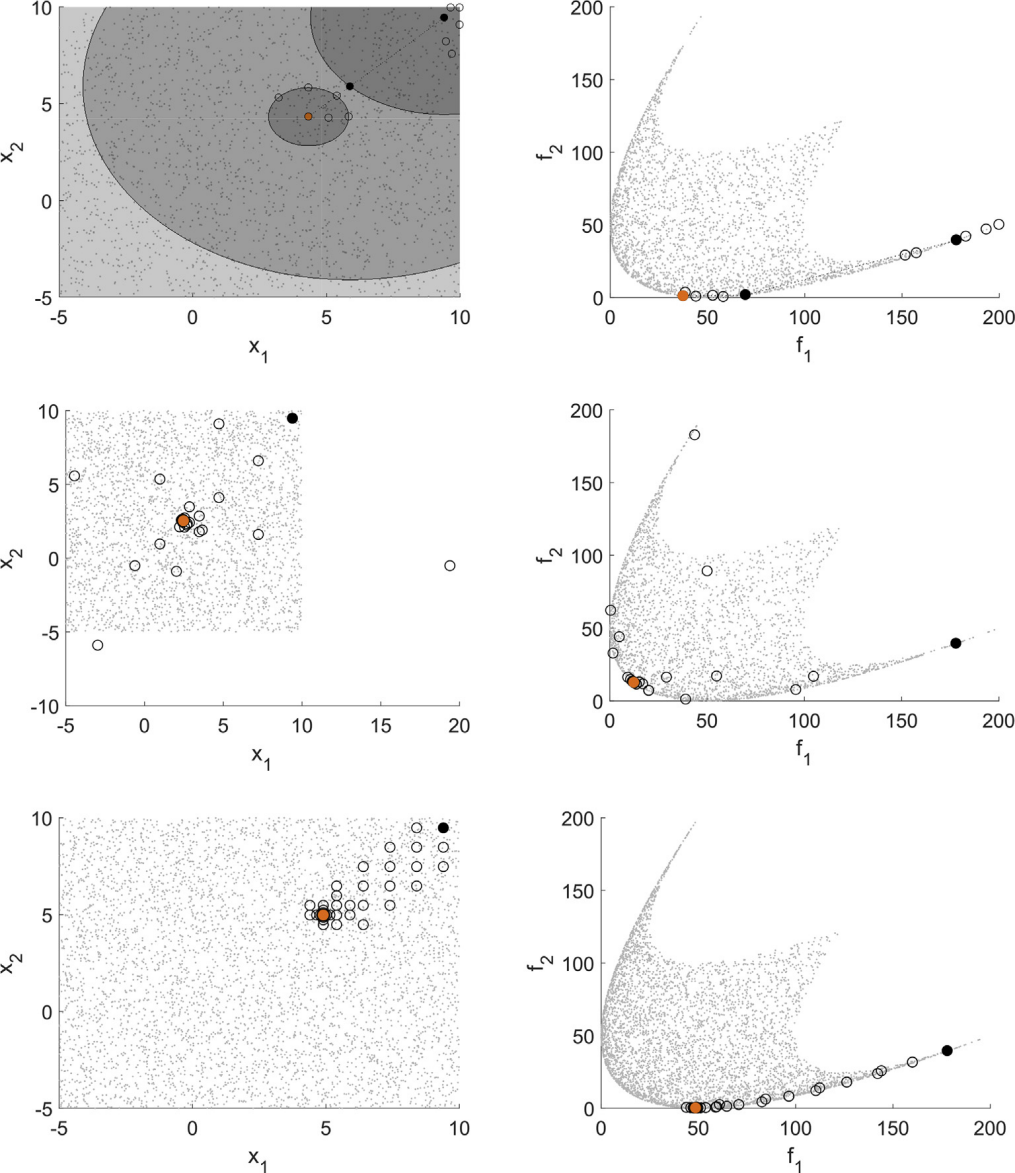
Test run for (BK1) for MHT (top), EFOS (middle) and DMS (bottom).

Fig. 1 shows one test result for MHT (domain top left, image space top right), EFOS (domain middle left, image space middle right) and DMS (domain bottom left, image space bottom right). The domain resp. image set is represented by scattered gray points, the starting point is marked black and the solution is marked orange. For MHT the iteration points are marked black and connected by a dotted line, the interpolation points that are evaluated to compute the model functions are marked as unfilled circles. In the domain the trust regions are depicted as gray shaded, transparent circles (the more areas overlap, the darker the gray shade). For EFOS and DMS it is not possible to distinguish between iteration points and further evaluated points during the iterations. Thus, all points evaluated for computing the solution are marked as unfilled circles and only the starting point and the solution are highlighted as for MHT.

For quadratic functions the quadratic interpolation model used for the expensive function in MHT is exact. Thus, the model built in the beginning of the algorithm is reused in all following iterations. Only in the last iteration, when it is checked if the iteration point is a Pareto critical point, the model function is recomputed in a local area. This can be seen in the top left part of Fig. 1, where the interpolation points are situated in the first and in the last trust region. The interpolation points are also close to the iteration points in the image space which the top right figure shows. Both figures illustrate the local search strategy of MHT.

EFOS computes more points than MHT and they are more spread both over the image space and the domain. During the run even infeasible points are generated. This can be seen only in the domain (middle left) since for illustrative reasons we used the same range in the image space for all figures on the right.

**Fig. 2:**
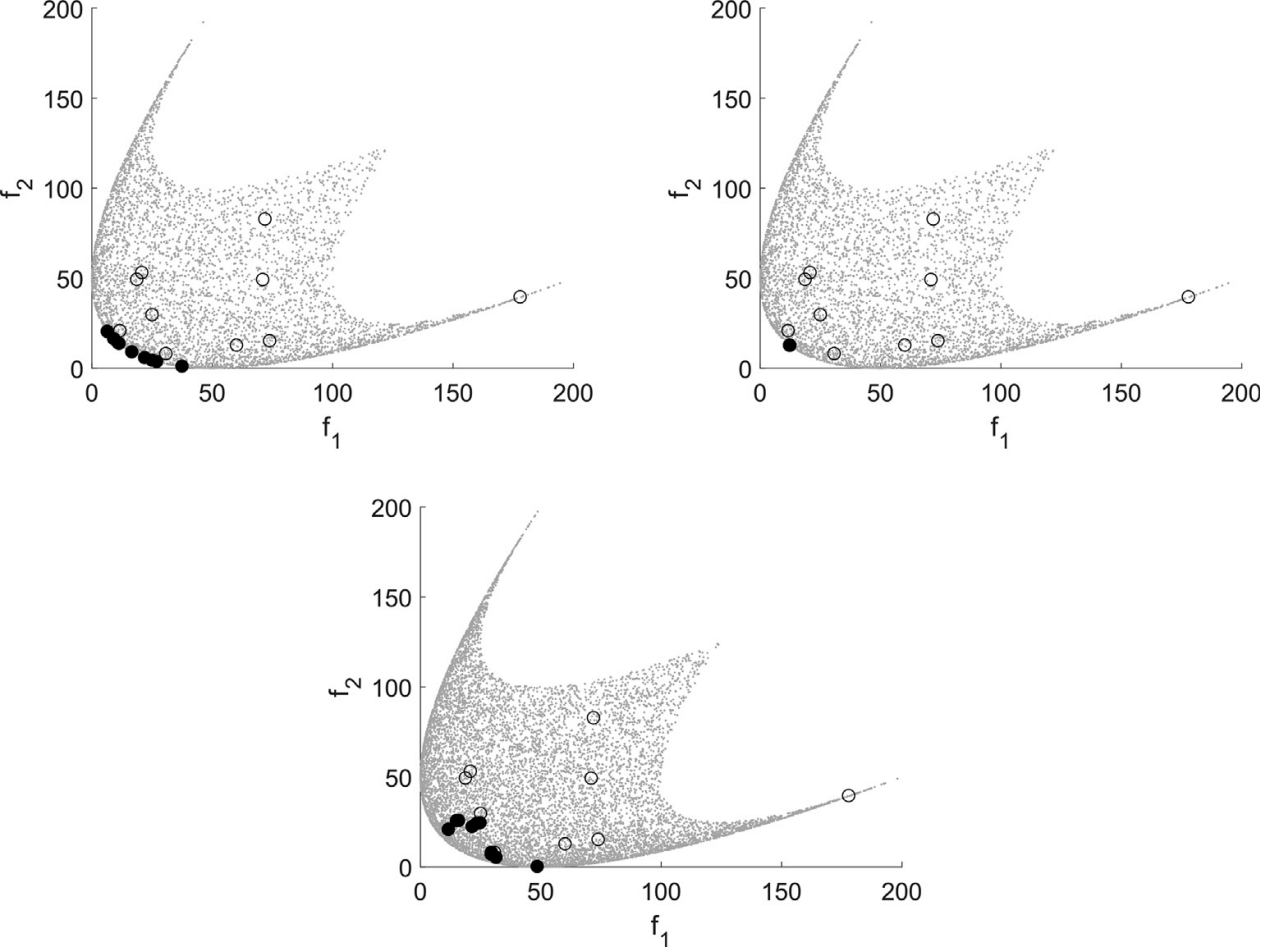
Multistart approach for (BK1) for MHT (top left), EFOS (top right) and DMS (bottom).

DMS also computes more points than MHT, but they are not spread over the domain as for EFOS, but accumulate in a local area. Apart from this, the bottom left figure illustrates the search along the coordinate directions. In the last iterations the step size decreases. In one run of MHT one Pareto critical point is computed. In general, different starting points generate different Pareto critical points due to the search strategy. A multistart approach with randomly generated starting points is illustrated in Fig. 2. The starting points are marked as unfilled circles and the obtained points are marked black. Fig. 2 shows that MHT (top left) and DMS (bottom) compute different nondominated points, whereas EFOS (top right) generates only one nondominated point. A reason for this is the weighted sum approach. Furthermore, not all resulting points from DMS are efficient points, some have still a large distance to the Pareto front.

As second test example we consider the convex, but not quadratic problem (T6) given by

(T6) $$\underset{x\in \text{Ω}}{\text{min}}\left(\begin{array}{c} f_{1}\left(x\right)\\ f_{2}\left(x\right) \end{array}\right)=\underset{x\in (0,100]{x\in (0,100]}^{2}}{\text{min}}\left(\begin{array}{c} -\text{ln}\left(x_{1}\right)-\text{ln}\left(x_{2}\right)\\ x_{1}^{2}+x_{2} \end{array}\right).$$

**Fig. 3:**
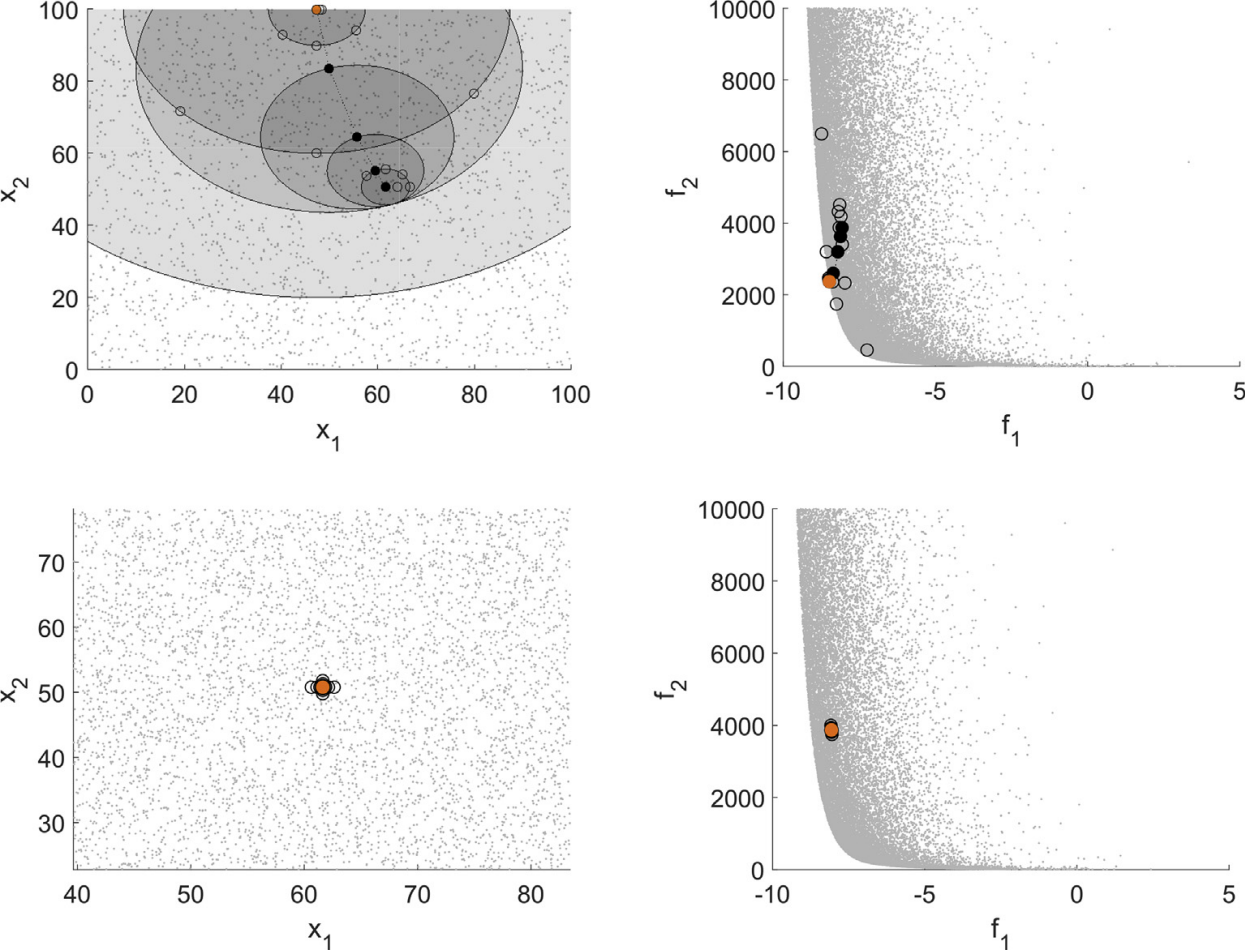
Test run for (T6) for MHT (top) and DMS (bottom).

For all test instances of this optimization problem EFOS is prematurely canceled due to an internal error. Consequently, we only show results for DMS and MHT. The as expensive declared function $f_{1}$ is not quadratic and therefore the interpolation model used in MHT is not exact. Though, the algorithm reuses old model information as often as possible which is illustrated for one specific instance, see the top left (domain) and top right image (image space) in Fig. 3. The bottom left and right image show the result for DMS in the domain and the image space with the same starting point.

**Fig. 4:**
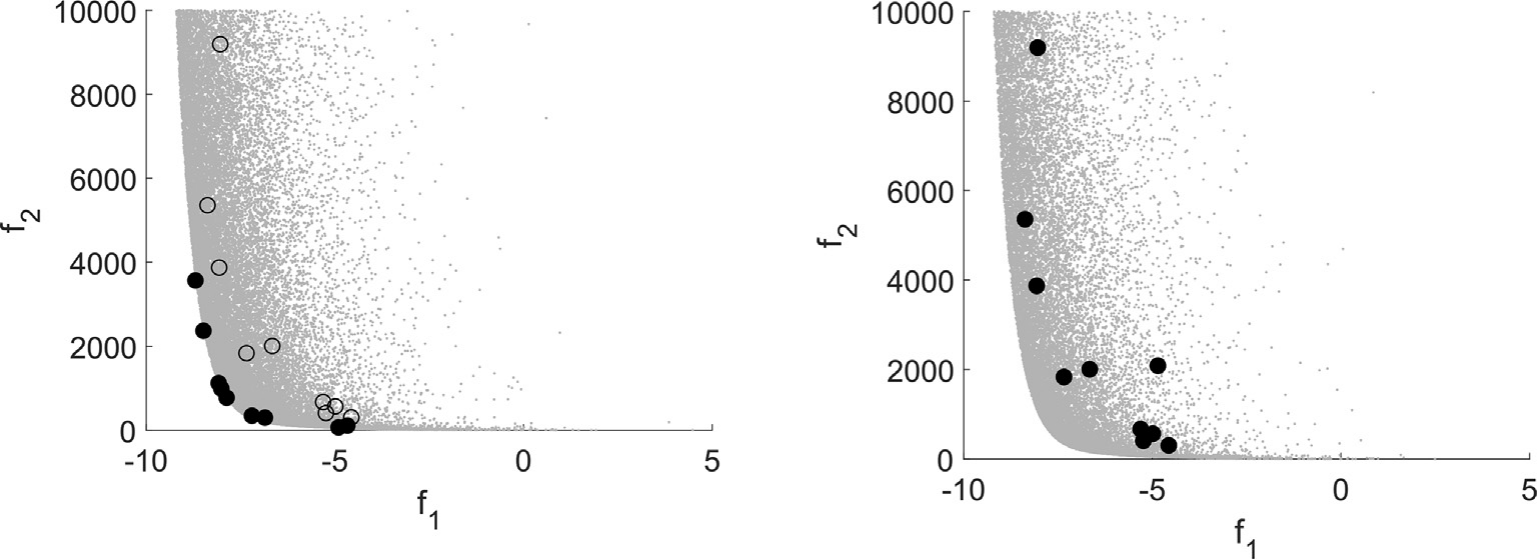
Multistart approach for (T6) for MHT (left) and DMS (right).

For this test instance the iterates of MHT move towards an efficient point with few interpolation points that are mostly close to the iteration points. As the top left figure shows, the model was only updated in some iterations and already evaluated points could be reused. Within 19 function evaluations of $f_{1}$ an efficient point is generated. In contrast, DMS terminates after 41 function evaluations with a point close to the starting point which is not close to an efficient point. The results of runs with randomly generated starting points are depicted in Fig. 4.

MHT generates well distributed nondominated points within 8–114 function evaluations. DMS terminates for all starting points with points close to them. This is illustrated in the right figure as all the unfilled starting points are overlapped by the filled points computed by DMS. All of these points are computed within 41 function evaluations and most of them are not close to the Pareto front.

**Fig. 5:**
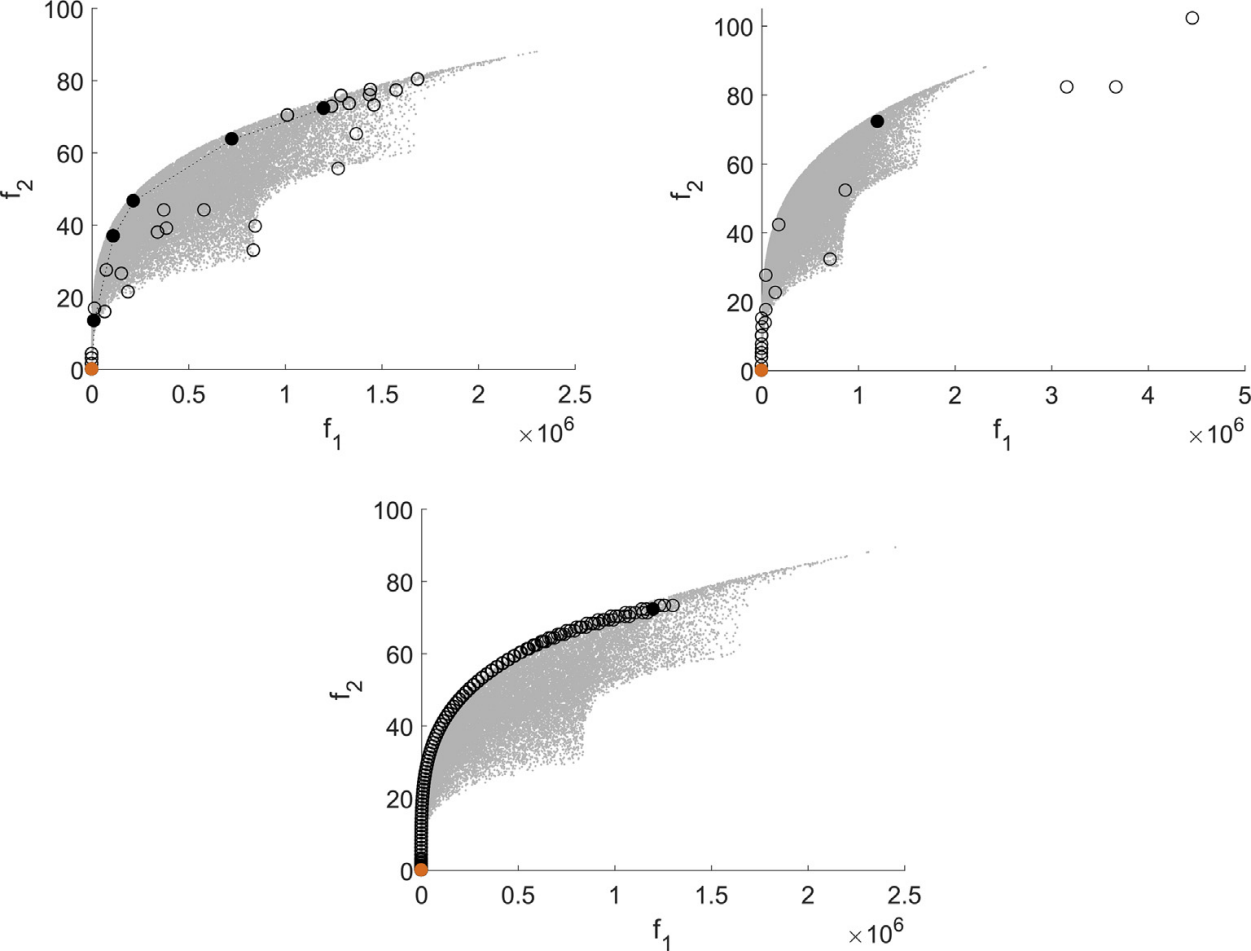
Test run for (T7) for MHT (top left), EFOS (top right) and DMS (bottom).

Another convex problem is (T7), but with a three-dimensional domain, given by

(T7) $$\underset{x\in \text{Ω}}{\text{min}}\left(\begin{array}{c} f_{1}\left(x\right)\\ f_{2}\left(x\right) \end{array}\right)=\underset{{x\in \left[0,30\right]}^{3}}{\text{min}}\left(\begin{array}{c} \overset{n}{\underset{i=1}{\sum }}x_{i}^{4}+\overset{n}{\underset{i=1}{\sum }}x_{i}^{3}\\ \overset{n}{\underset{i=1}{\sum }}x_{i} \end{array}\right)$$

and with $f_{1}$ declared as expensive function. The unique efficient point for this optimization problem is $\bar{x}={\left(0,0,0\right)}^{⊤}$ with the function values $f_{1}\left(\bar{x}\right)=f_{2}\left(\bar{x}\right)=0$. For all considered starting points all three algorithms compute this unique nondominated point respectively a point with vanishing distance to it. This is shown in Fig. 5 for one instance.

The range of function evaluations for all instances is similar for MHT (29–57) and EFOS (9–75). Compared to DMS with 206–348 evaluations, both MHT and EFOS save function evaluations. The reason for this large difference is the direct search approach of DMS which produces, as depicted in Fig. 5 for one instance, dense evaluated points in the image space. Again, EFOS computes also infeasible points during the runs which is also the case for the instance depicted in Fig. 5.

#### 2.2.2 Nonconvex test problems

MHT is a local method and as proved in Ref. [13], the accumulation points of the generated sequence satisfy, in case several assumptions are fulfilled, a necessary condition for local weak efficiency. For convex problems local and global optimality is identical. For nonconvex problems this is in general not the case. In the following, we report on how the algorithm performs for nonconvex problems and exemplarily show the results of three nonconvex test problems.

**Fig. 6:**
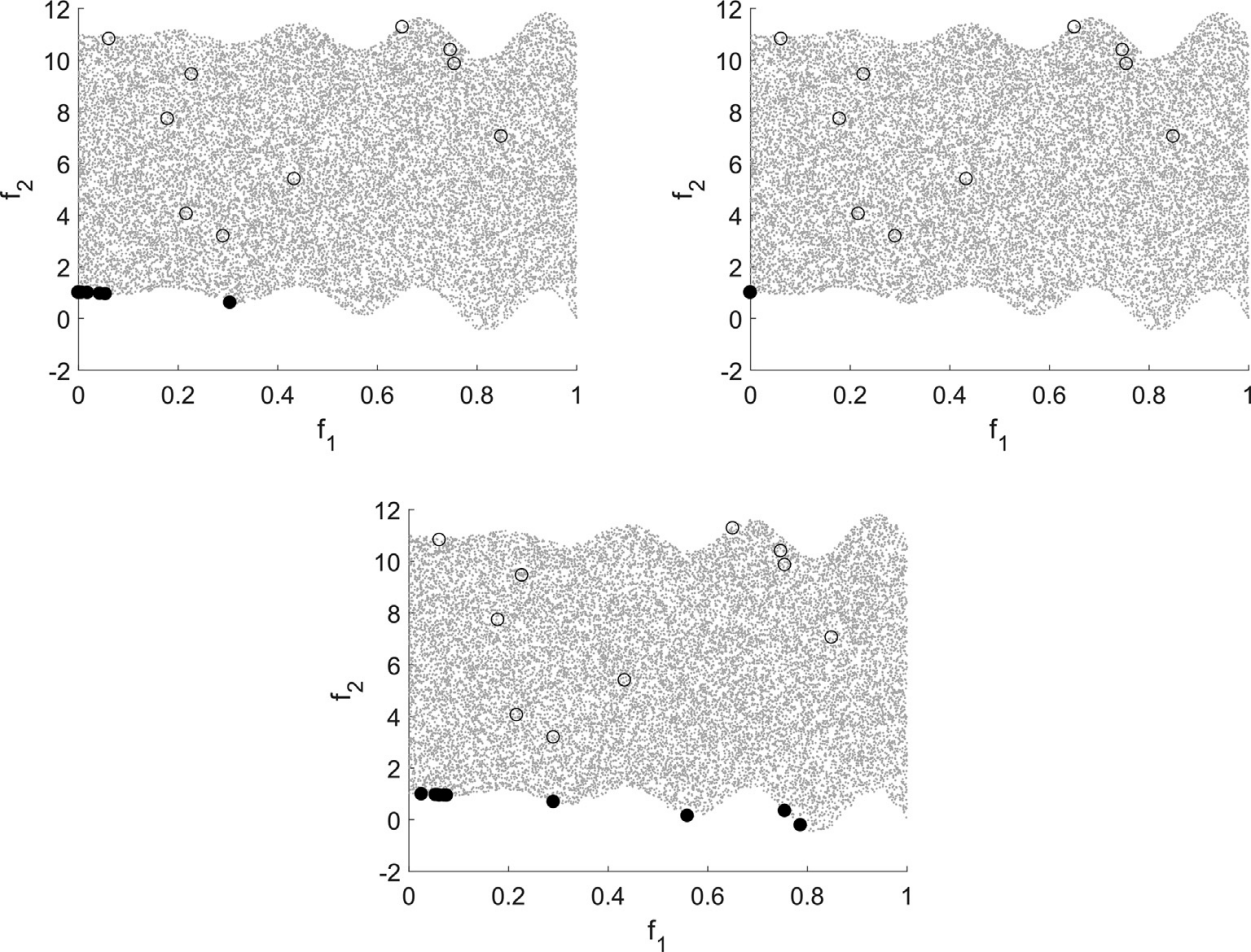
Multistart approach for (Deb513) for MHT (top left), EFOS (top right) and DMS (bottom).

The first one is (Deb513) from Refs. [1], [2] defined by

(Deb513) $$\underset{x\in \text{Ω}}{\text{min}}\left(\begin{array}{c} f_{1}\left(x\right)\\ f_{2}\left(x\right) \end{array}\right)=\underset{{x\in \left[0,1\right]}^{2}}{\text{min}}\left(\begin{array}{c} x_{1}\\ g\left(x\right)h\left(x\right) \end{array}\right)$$

with $g\left(x\right)=1+10x_{2}$, $h\left(x\right)=1-{\left(x_{1}/g\left(x\right)\right)}^{2}-\left(x_{1}/g\left(x\right)\right)\text{sin}\left(8\pi x_{1}\right)$ and a disconnected Pareto front. For this test problem $f_{2}$ is declared as expensive function. All three algorithms are capable of computing a nondominated point for all instances of this test problem as Fig. 6 shows. Furthermore, it shows that again MHT (top left) and DMS (bottom) generate several nondominated points whereas EFOS (top right) computes only one nondominated point.

**Fig. 7:**
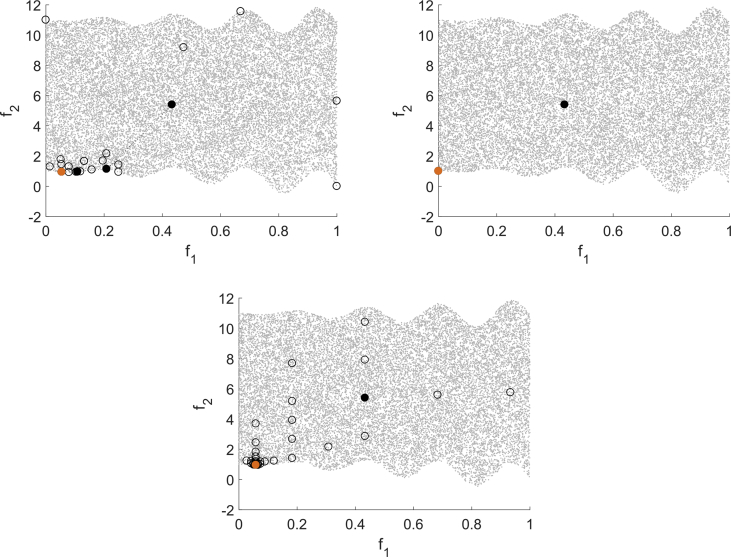
Test run for (Deb513) for MHT (top left), EFOS (top right) and DMS (bottom).

The different search strategies are recognizable in Fig. 7 which shows the result of one run with the same starting point for all three algorithms. EFOS (top right) computes the individual minimum of function $f_{1}$ within 7 function evaluations for all starting points. This is due to the weighted sum approach. The points that are evaluated during the run are mostly infeasible and situated far outside the pictured area. We zoomed in for reasons of illustration and comparison.

For MHT (25 function evaluations) the top left figure illustrates the local search behavior controlled in the image space. Only for the initial model the interpolation points are spread broader over the image space, yet in the further iterations the interpolation points are close to the iteration points. The direct search approach of DMS (38 function evaluations) is visible in the bottom figure. It illustrates the search along the axes. DMS and MHT need a similar amount of function evaluations, yet both need significantly more than EFOS.

**Fig. 8:**
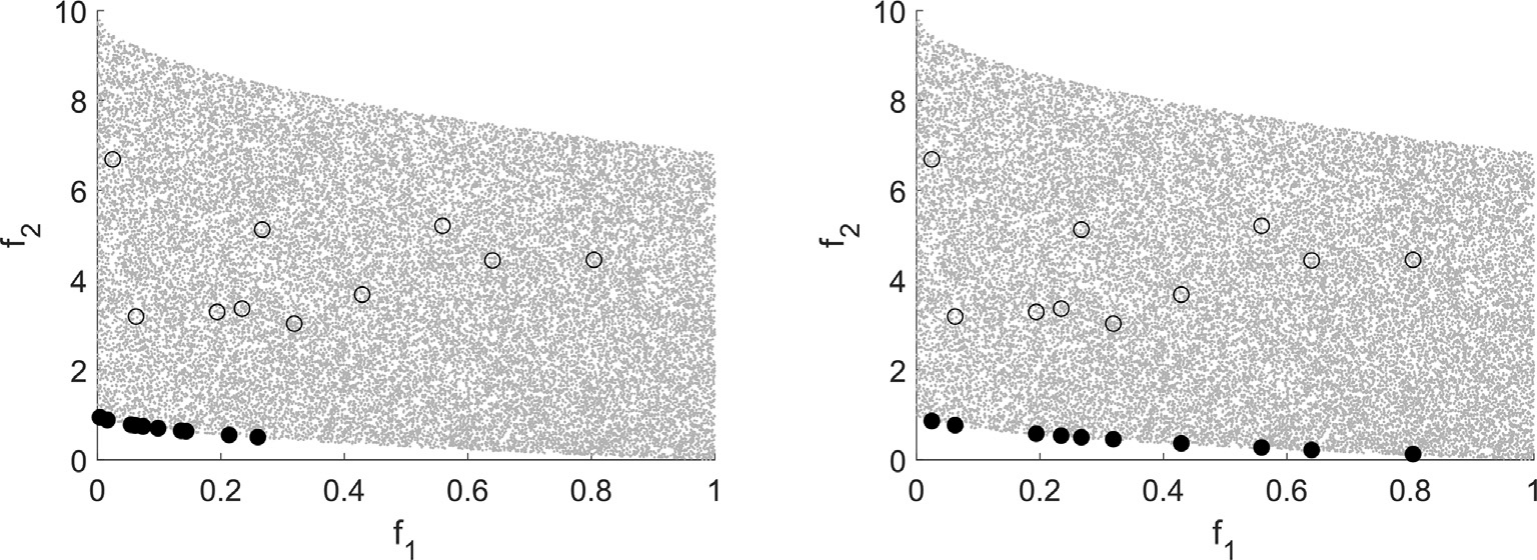
Multistart approach for (Deb513) for MHT (left) and DMS (right).

As second noncovex test problem we consider (Jin2) with n=4 from Refs. [1], [9] defined by

(Jin2) $$\underset{x\in \text{Ω}}{\text{min}}\left(\begin{array}{c} f_{1}\left(x\right)\\ f_{2}\left(x\right) \end{array}\right)=\underset{{x\in \left[0,1\right]}^{4}}{\text{min}}\left(\begin{array}{c} x_{1}\\ g\left(x\right)\left(1-\sqrt{\frac{x_{1}}{g\left(x\right)}}\right) \end{array}\right)$$

with $g\left(x\right)=1+3\sum_{i=2}^{4}x_{i}$ and $f_{2}$ declared as expensive. EFOS could not compute an efficient solution for this test problem, but stopped with an internal error for the considered starting points. DMS and MHT compute nondominated points for all instances of this problem. The multistart approach with randomly chosen starting points in Fig. 8 shows that both compute different nondominated points given different starting points. However, the points computed by DMS are better spread than the points computed by MHT. Regarding the required function evaluations no clear statement can be made which algorithm needs less (DMS 92–126, MHT 30–169).

**Fig. 9:**
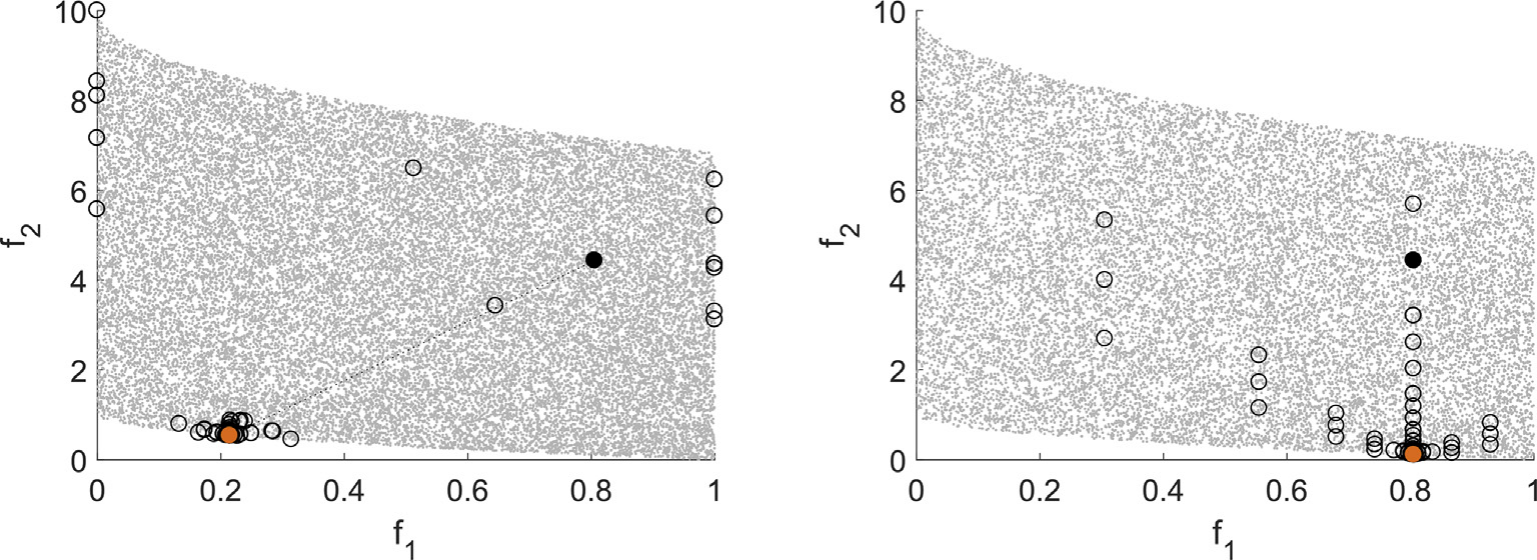
Test run for (Jin2) for MHT (left) and DMS (right).

In all runs MHT needs many function evaluations in the end of the procedure. Due to the nonconvexity and the local search strategy this number of function evaluations is needed to ensure the stopping criterion being fulfilled. This is exemplarily shown for one specific run in Fig. 9. This instance again illustrates the coordinate search of DMS.

As last nonconvex test problem we consider (FF) with n=3 from Ref. [5] given by

(FF) $$\underset{x\in \text{Ω}}{\text{min}}\left(\begin{array}{c} f_{1}\left(x\right)\\ f_{2}\left(x\right) \end{array}\right)=\underset{{x\in \left[-4,4\right]}^{3}}{\text{min}}\left(\begin{array}{c} 1-\text{exp}\left(-\overset{n}{\underset{i=1}{\sum }}{\left(x_{i}-\frac{1}{\sqrt{n}}\right)}^{2}\right)\\ 1-\text{exp}\left(-\overset{n}{\underset{i=1}{\sum }}{\left(x_{i}+\frac{1}{\sqrt{n}}\right)}^{2}\right) \end{array}\right)$$

with $f_{1}$ declared as expensive function. This test problem illustrates that Pareto criticality is only a necessary condition for local efficiency and that MHT does not necessarily generate an efficient point if it stops legitimately according to the stopping criterion.

**Fig. 10:**
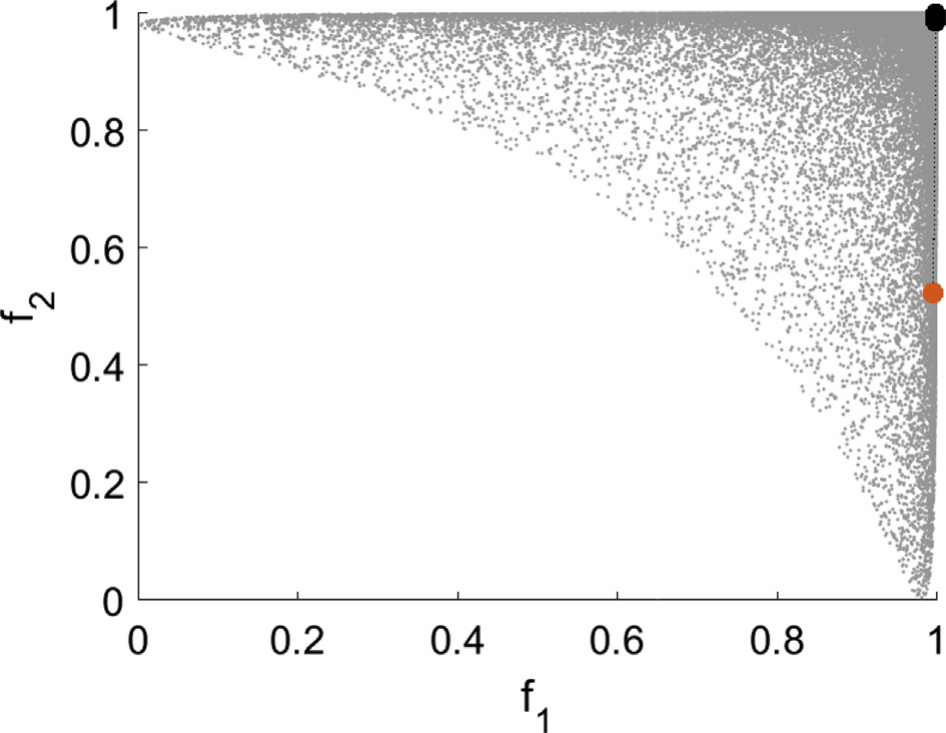
Test run for (FF) for MHT.

Fig. 10 shows the result of MHT for one test instance of (FF). The algorithm terminates with the point $\bar{x}={\left(-0.0604,-1.2138,-0.8433\right)}^{⊤}$ with the function values $f_{1}\left(\bar{x}\right)=0.9964$ and $f_{2}\left(\bar{x}\right)=0.5234$. The stopping criterion indicates that $\bar{x}$ is Pareto critical, since the step size $\bar{t}$ of the auxiliary Pascelotti-Serafini problem that is used in MHT is small enough($\bar{t}=3.319810^{-7}$). For further details see Section 3.2 and Section 5.1 in [13].

Additionally, another criterion using an auxiliary function denoted by *ω* and described later in subsection 2.2.4 confirms that $\bar{x}$ is a Pareto critical point ($\omega \left(\bar{x}\right)=8.260410^{-3}$). Though, this point is not an efficient point as the illustration in the image space in Fig. 10 shows.

#### 2.2.3 Scalable test problems

We considered test problems with different dimensions for the domain and did also runs for MHT with two scalable test problems up to dimension 50. As expected the numerical effort rises when the dimension rises. The higher the dimension is, the more function evaluations are required to build up a model. If in every iteration a complete new set of interpolation points would be computed, a total number of (n+1)(n+2)/2 function evaluations would be needed for the expensive function in every iteration. However, in MHT the model is not updated in every iteration, but only if necessary and if it is updated, the former interpolation points are reused if possible. Thus, the number of new function evaluations necessary is kept to a minimum.

Furthermore, we suggest in Ref. [13] to use for higher dimensions a linear interpolation model also based on Lagrange polynomials. This needs only n+1 interpolation points and therefore the number of function evaluations also reduces. Though, a linear model needs to be updated more often since it is less accurate. Table 2 gives an overview of how many function evaluations are required to compute one model function (quadratic/linear interpolation with Lagrange polynomials) depending on the dimension of the domain.

**Table 2:** Function evaluations for computing one model function.

n	2	3	4	5	10	20	30	40	50
quadratic model	6	10	15	21	66	231	496	861	1326
linear model	3	4	5	6	11	21	31	41	51

**Fig. 11:**
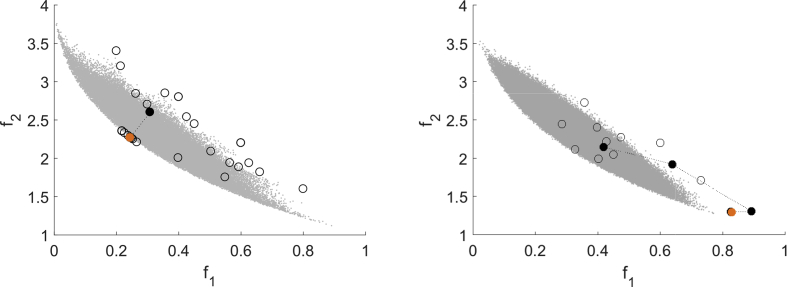
Test run for (Jin1) for MHT with n=5 (left) and n=10 (right).

In the numerical tests we used the linear models for all instances with dimension n≥10. To illustrate the behavior of MHT with rising dimension we consider the scalable test problem (Jin1) from Refs. [1], [9] defined by

(Jin1) $$\underset{x\in \text{Ω}}{\text{min}}\left(\begin{array}{c} f_{1}\left(x\right)\\ f_{2}\left(x\right) \end{array}\right)=\underset{{x\in \left[0,1\right]}^{n}}{\text{min}}\left(\begin{array}{c} \frac{1}{n}\overset{n}{\underset{i=1}{\sum }}x_{i}^{2}\\ \frac{1}{n}\overset{n}{\underset{i=1}{\sum }}{\left(x_{i}-2\right)}^{2} \end{array}\right)$$

with $f_{1}$ declared as expensive. It has been tested with MHT for n∈{2,3,4,5,10,20,30,40, 50}. Fig. 11 shows runs with MHT for n=5 on the left and n=10 on the right.

**Fig. 12:**
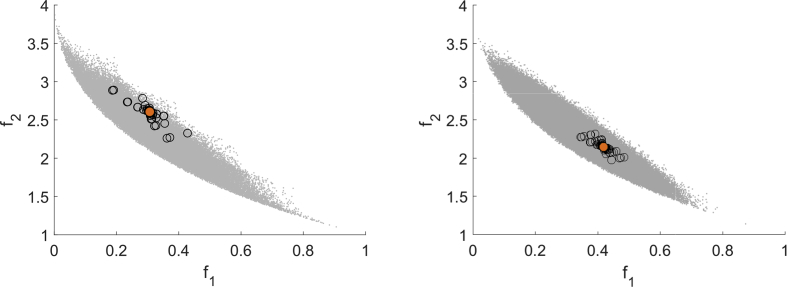
Test run for (Jin1) for DMS with n=5 (left) and n=10 (right).

For n=10 a linear model is used which as expected worsens the predictions of the model functions. Since the trial point acceptance test in MHT does not demand a strict decrease in every component, but is instead a weaker formulation, also points that increase one of the objective functions can be accepted. Fig. 12 shows the results for DMS applied to (Jin1) with n=5 and n=10 with the same starting points as used in the runs depicted in Fig. 11. MHT needs 42 function evaluations for n=5 (21 for n=10) and therefore significantly less than DMS which needs 78 evaluations (152 for n=10). . Even though DMS computes many function values, it explores only the area close to the starting point and terminates with a point close to the starting point.

In the following we give a short overview of the number of function evaluations required by MHT and DMS for the scalable test examples (Jin1) and T4, see Test Problem 4 in section 3. Table 3 gives an overview of the range (R) and mean value (M) of used function evaluations for MHT.

**Table 3:** Function evaluations (range and mean value) per dimension for MHT.

n	2	3	4	5	10	20	30	40	50
Jin1 (R)	11–12	20–32	15–46	42–82	15–667	46–621	39–322	63–244	109–316
Jin1 (M)	11.4	24.3	29.9	52.4	107.3	174.4	120.2	121.3	165
T4 (R)	12–14	21–22	31–32	43–44	68–206	252–433	399–561	609–1013	1023–1459
T4 (M)	13.2	21.4	31.4	43.7	152.1	338	483.7	794.4	1246.9

As already seen in the instance of (Jin1) presented above, the ranges in Table 3 show that there are instances for which a higher dimension required less function evaluations than a lower dimension, e.g. dimension 5 and 10. This is due to the choice of starting points and due to the different kinds of model functions. From dimension 10 onwards linear model functions are used for the as expensive declared objective function. Along with the choice of a starting point and the local search strategy this can cause a lower total number of function evaluations for single instances. However, in general, the tendency of rising function evaluations with rising dimension is apparent.

The comparison method DMS is a direct search approach from Ref. [1]. For the general direct search approach 2n function evaluations are needed in every iteration. Table 4 gives an overview of this number up to dimension 50.

**Table 4:** Function evaluations per iteration for general direct search.

n	2	3	4	5	10	20	30	40	50
eval. per it.	4	6	8	10	20	40	60	80	100

For DMS as it is implemented and available, Table 5 shows the range (R) and mean value (M) of function evaluations needed for the two scalable test problems. We set the maximum number of allowed function evaluations to 2000. This is not enough for some instances of T4 as Table 5 shows. For these instances DMS terminated with the maximum number of function evaluations reached without having computed an efficient point.

**Table 5:** Function evaluations (range and mean value) per dimension for DMS.

n	2	3	4	5	10	20	30	40	50
Jin1 (R)	30–35	47–52	63–69	78–85	152–165	307–328	469–488	622–648	767–818
Jin1 (M)	33.1	49.8	66.2	81.7	160.7	321.4	479.7	637.2	800.5
T4 (R)	54–152	71–213	117–424	181–510	706–1128	1849–2000	2000	2000	2000
T4 (M)	83.8	133.2	212.1	303.5	834.1	1998.6	2000	2000	2000

Table 3, Table 5 give a first impression of how the dimension *n* influences MHT in comparison to DMS. As expected, the direct search approach needs significantly more function evaluations with rising dimension than MHT. The mean values of function evaluations per dimension for all instances of the scalable test problems FF, Jin1, Jin2, Jin3, Jin4, T4 (see Table 1 in subsection 2.1) are listed in Table 6.

**Table 6:** Mean value of function evaluations per dimension for all scalable problems for MHT and DMS.

n	2	3	4	5	10	20	30	40	50
MHT	16.09	39.97	72.63	109.22	129.7	256.2	301.95	457.85	705.95
DMS	46.40	79	122.38	174.52	834.10	1153.2	1239.8	1318.6	1400.3

This indicates that, compared to the direct search approach of DMS, the algorithm introduced in Ref. [13] can also save function evaluations in higher dimensions. However, it is important to note that DMS does not make use of any derivative information, also not of the cheap function. Therefore, it must be expected that DMS needs more function evaluations than any method that does use such information.

#### 2.2.4 Performance profiles

**Fig. 13:**
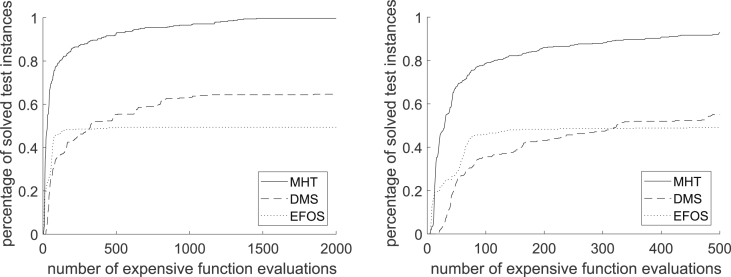
Performance Profile for MHT, DMS and EFOS for 348 convex instances, cf. Fig. 2 in Ref. [13].

For classifying the test runs as successful or not successful the distance to the Pareto front, the set of nondominated points, is used. If it falls below a problem dependent constant, the test run for an instance is classified as solved. To compare the performance of the algorithms, the number of function evaluations for the as expensive declared function is counted until the algorithm terminates. Fig. 13 Figure 2 in [13] shows a performance profile for all 348 convex test instances in full range on the left and zoomed in on the right.

If up to 480 function evaluations are allowed for the expensive function, DMS and EFOS behave similar. With further function evaluations DMS is capable of solving further test instances whereas EFOS stagnates and cannot solve more instances.

**Fig. 14:**
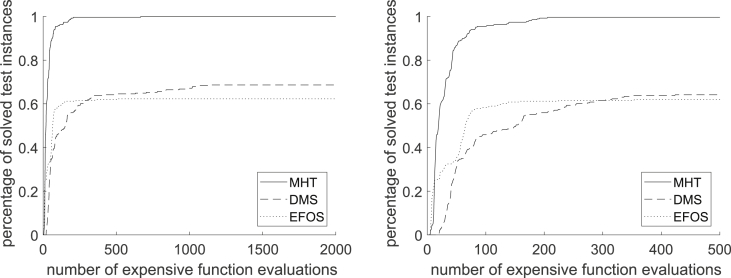
Performance Profile for MHT, DMS and EFOS for 268 convex instances.n≤10

In general, Fig. 13 shows that MHT needs less function evaluations than EFOS and DMS to solve the convex test problems. It solves all 348 convex test instances within at most 1459 expensive function evaluations. This high number is due to the high dimensional test instances included. If considering only test instances up to dimension 10 all convex instances are solved by MHT after 667 expensive function evaluations as the performance profile in Fig. 14 shows (full range left, zoomed in right). With the same amount of function evaluations (667) DMS solves 64.93% and EFOS 62.31% of the convex test problems up to dimension 10.

As already noted, MHT is a local method and generates a sequence of iteration points. The accumulation points of this sequence fulfill, in case several assumptions are fulfilled, a necessary condition for local weak efficiency (Pareto criticality). The fact that Pareto criticality is a necessary condition for local optimality must be regarded when considering nonconvex problems since local optimality is in general not synonymous to global optimality. If the algorithm stops legitimately in a Pareto critical point, the distance to the Pareto front does not need to converge to zero. Thus, for a performance profile over all considered test examples we do not only use this distance to classify test instances as solved, but complement it with a measure for Pareto criticality. The auxiliary function $\omega :R^{n}\to R$ defined by

$$\omega \left(x\right):=-\underset{\left\|d\right\|\leq 1}{\text{min}}\underset{i=1,\ldots ,q}{\text{max}}\nabla_{x}f_{i}{\left(x\right)}^{⊤}d$$

for $f_{i}:R^{n}\to R$ continuously differentiable functions, i=1,…,q, characterizes Pareto criticality. According to Ref. [4] *ω* is a continuous function, it holds ω(x)≥0 for all $x\in R^{n}$ and a point $x\in R^{n}$ is Pareto critical for *(MOP)* if and only if it holds ω(x)=0.

Consequently, given $\bar{x}$ the solution generated by one of the considered algorithms (MHT, DMS, EFOS), we classify a test instance as solved if either the distance of $f\left(\bar{x}\right)$ to the Pareto front is small enough or if it holds $\omega \left(\bar{x}\right)\leq \varepsilon$. We chose ε=0.1 for the data analysis.

**Fig. 15:**
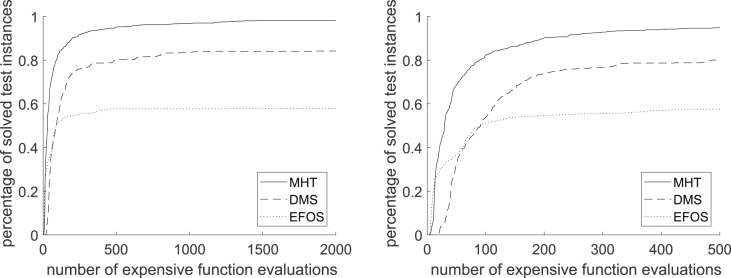
Performance Profile for MHT, DMS and EFOS for all 802 instances.

Using these classifications, the performance profile in Fig. 15 shows how many of all 802 considered test instances are solved depending on the required function evaluations for MHT, DMS and EFOS. The full range is shown on the left and on the right the performance profile is zoomed in to 500 function evaluations.

Fig. 15 illustrates that by applying MHT 98.13% of all test instances are solved with either an efficient or a Pareto critical point. Within the same number of function evaluations (1459) EFOS solved 57.98% and DMS solved 84.04% of all considered test instances. Thus, MHT solved more test problems than both comparison methods and needs less function evaluations. Although the behavior of DMS is similar to our method, still MHT saves computation time and solves more instances in terms of distance to the Pareto front or Pareto criticality.

## Appendix A Self-chosen Test Problems

In the following we formulate the objective functions and the constraint sets of the self-chosen test examples (T1)–(T8) listed in Table 1.

**Test Problem 1** (T1). *(MOP) with* n=2*,* q=2*, constraint set* $\text{Ω}=R^{2}$*,*

$$\begin{array}{l} f_{1}\left(x\right)=\frac{1}{2}x_{1}^{2}+x_{2}^{2}-10x_{1}-100\\ f_{2}\left(x\right)=x_{1}^{2}+\frac{1}{2}x_{2}^{2}-10x_{2}-100 \end{array}$$

and $f_{1}$ declared as expensive.

**Test Problem 2** (T2). (MOP) with n=2, q=2, constraint set $\text{Ω}=R^{2}$,

$$\begin{array}{l} f_{1}\left(x\right)=\text{sin}x_{2}\\ f_{2}\left(x\right)=1-\text{exp}\left(-{\left(x_{1}-\frac{1}{\sqrt{2}}\right)}^{2}-{\left(x_{2}-\frac{1}{\sqrt{2}}\right)}^{2}\right) \end{array}$$

and $f_{2}$ declared as expensive.

**Test Problem 3** (T3). *(MOP) with* n=2*,* q=2*, constraint set* ${\text{Ω}=\left[-2,2\right]}^{2}$*,*

$$\begin{array}{l} f_{1}\left(x\right)=x_{1}+2\\ f_{2}\left(x\right)=x_{1}-2+x_{2} \end{array}$$

and $f_{2}$ declared as expensive.

**Test Problem 4** (T4). *(MOP) with* n∈{2,3,4,5,10,20,30,40,50}*,* q=2*, constraint set* ${\text{Ω}=\left[-10,10\right]}^{n}$*,*

$$\begin{array}{l} f_{1}\left(x\right)=\overset{n-1}{\underset{i=1}{\sum }}x_{i}^{2}+2\\ f_{2}\left(x\right)=\overset{n}{\underset{i=1}{\sum }}x_{i}-2 \end{array}$$

and $f_{1}$ declared as expensive.

**Test Problem 5** (T5). *(MOP) with* n=2*,* q=2*, constraint set* Ω=(0,30]×[0,30]*,*

$$\begin{array}{l} f_{1}\left(x\right)=x_{1}\text{ln}\left(x_{1}\right)+x_{2}^{2}\\ f_{2}\left(x\right)=x_{1}^{2}+x_{2}^{4} \end{array}$$

and $f_{1}$ declared as expensive.

**Test Problem 6** (T6). *(MOP) with* n=2,q=2*, constraint set* ${\text{Ω}=(0,100]}^{2}$*,*

$$\begin{array}{l} f_{1}\left(x\right)=-\text{ln}\left(x_{1}\right)-\text{ln}\left(x_{2}\right)\\ f_{2}\left(x\right)=x_{1}^{2}+x_{2} \end{array}$$

and $f_{1}$ declared as expensive.

**Test Problem 7** (T7). *(MOP)with* n=3,q=2*, constraint set* ${\text{Ω}=\left[0,30\right]}^{3}$*,*

$$\begin{array}{l} f_{1}\left(x\right)=\overset{n}{\underset{i=1}{\sum }}x_{i}^{4}+\overset{n}{\underset{i=1}{\sum }}x_{i}^{3}\\ f_{2}\left(x\right)=\overset{n}{\underset{i=1}{\sum }}x_{i} \end{array}$$

and $f_{1}$ declared as expensive.

**Test Problem 8** (T8). *(MOP) with* n=3,q=3*, constraint set* Ω=(0,10]×[0,10]×[0,10]

$$\begin{array}{l} f_{1}\left(x\right)=\overset{n}{\underset{i=1}{\sum }}x_{i}^{3}\\ f_{2}\left(x\right)=\overset{n-1}{\underset{i=1}{\sum }}{\left(x_{i}-4\right)}^{2}+x_{n}^{2}\\ f_{3}\left(x\right)=-\text{ln}\left(x_{1}\right)+5\overset{n}{\underset{i=2}{\sum }}x_{i}^{2} \end{array}$$

and $f_{3}$ declared as expensive.
